# Understanding the conversation around COVID-19 and eating disorders: A thematic analysis of Reddit

**DOI:** 10.1186/s40337-022-00530-z

**Published:** 2022-01-15

**Authors:** Ashleigh N. Shields, Elise Taylor, Jessica R. Welch

**Affiliations:** 1 Northeastern University, Boston, MA USA; 2 Purdue University, West Lafayette, USA; 3 Embry-Riddle Aeronautical University, Daytona Beach, USA

**Keywords:** COVID-19, Eating disorders, Reddit, Thematic analysis

## Abstract

**Background:**

Current research has found dramatic changes in the lives of those with eating disorders (EDs) during the COVID-19 pandemic. We build on existing research to investigate the long-term effects and adaptations that people with EDs have faced due to COVID-19 related changes.

**Method:**

We collected 234 posts from three separate time periods from the subreddit r/EatingDisorders and analyzed them using thematic analysis. The posts were examined for initial patterns, and then those concepts were grouped into themes to reveal the authentic experiences of people living with EDs during the COVID-19 pandemic.

**Results:**

Initially, we found “lack of control” and “familial influences (loved ones seeking support)” emerge as themes within our broader data set throughout all three timeframes. There were additional themes that were present in only one or two of the collection periods. These themes consisted of “symptom stress,” “technical stresses and concerns,” and “silver linings.”

**Conclusions:**

Our analysis shows that people with EDs have fought significantly during the pandemic. Initially, the (lack of) control and routine in their lives has caused symptoms to become more challenging, while being forced to move back home also caused significant stress. However, concerns transformed as the pandemic progressed, resulting in new pressures causing people to exhibit novel ED symptoms or relapse altogether. Also notable is the relatively few COVID-specific posts as the pandemic progressed, suggesting that people have accepted COVID as their “new normal” and begun to build resilience to the challenges associated. These are vital factors for clinicians to consider as they begin taking existing and new patients, particularly as face-to-face treatment options become a possibility again.

**Plain English Summary:**

Existing research shows that the COVID-19 pandemic has transformed the lives of people who live with eating disorders in various ways. First, the pandemic has placed barriers on the path to recovery by limiting coping mechanism (and sometimes removing them altogether) and changing their relationships with food and the people in their lives. Second, the pandemic has forced treatment options to change since ED patients can no longer seek treatment face-to-face. Finally, there have been unexpected benefits to the pandemic, such as allowing individuals time to slow down and focus on their mental health. Previous studies examined individuals in clinical contexts rather than in their natural environments. We explored an online forum for people with eating disorders for the various themes that were discussed at three points over the period of March 2020-December 2020 and found that many people with EDs report worsening symptoms or relapse. However, we also noted that, compared to the beginning of the pandemic, people seemed to be less frequently asking for support during the third data collection period, implying an adaptation to the “new normal” of life in a pandemic. We conclude with a discussion of the findings.

## Background

The Pandemic Year(s)—2019–2021—fundamentally uprooted and transformed the lives of virtually every individual. No longer could people find social support in a hug when that hug could mean death. No more could one laugh or cry with a family member when those droplets of moisture could carry long-term illness. While many people were concerned about their safety regarding the physical symptoms of COVID-19, the mental impact has contributed to increased symptoms of anxiety, depression, and post-traumatic stress; in the United States alone, 37% of individuals exhibit signs of anxiety or depression [1–5]. Despite rising mental impact, the increased risks associated with COVID-19 made traditional support avenues, such as group therapies and individual provider visits, difficult or impossible. This has created a complex challenge for individuals with mental health issues. Individuals fighting eating disorders (EDs) are among the most impacted by this, as emerging research is beginning to show [3, 6].

### COVID-19 and EDs

Current research on the confluence of EDs and COVID-19 indicates that the impacts are wide-reaching and felt throughout the world, as one might expect [3–8]. Two main themes have emerged from the widespread impact: First, people are experiencing various barriers (including social, role, and support), and second, there are unexpected benefits for those battling EDs during the pandemic.

First, current research has indicated that the pandemic has caused wide-ranging social barriers amongst those fighting EDs, including isolation and changes in accountability/responsibility [9], and these social barriers often exacerbate existing negative behaviors. People experiencing stressful life events—such as a death in the family or a pandemic—experience a dramatic increase in harmful coping mechanisms and weight/food control behaviors, and [10]. As a result, people experiencing EDs have reported an increased usage of harmful coping mechanisms. Social isolation due to COVID-19 limits access to one’s support, which makes the patient more susceptible to the repercussions affiliated with stress [11]. Anxiety and depression have emerged as frequent, significant social barriers for people enduring the pandemic. Studies have indicated a tripling of the percentage of people who experience anxiety and/or depression and 53.8% of patients experienced moderate to severe symptoms [12, 13]. Social barriers related to COVID-19 are not unique to people living with EDs but, amongst a population with frequent comorbidities related to anxiety and depression, those issues are only exacerbated [14]. Researchers have also found that the rise in screen time during the pandemic contributed to increased anxiety due to increased media coverage related to gaining weight and exercising [7]. People living with EDs reported finding this near-constant media barrage of health, exercise, and food consumption to be triggering and harmful to their ongoing recovery, even resulting in ED symptoms remerging.

Second, COVID-19 has also caused role barriers to develop or be intensified. Individuals battling EDs tend to rediscover their identity during recovery, but lockdown created barriers to that process by limiting access to common aspects of a routine that may have proved vital to an individual pursuing recovery. This resulted in fewer strategies being helpful, a clouding of emotional insight, and a lack of acceptance for emotions [15]. An analysis of Reddit posts early in the pandemic found that participants' new living situations and daily activities resulted in decreased privacy, less resilient social support networks, and increased triggering situations as family took note of disordered behaviors. Additionally, they tended to have more ED symptomology as well as more adverse emotions (i.e., anxiety, fear, anger, guilt, loneliness, and depression) [16]. Similarly, another study found the worsening of ED symptomology was related to lower levels of self-directedness and ineffective coping strategies employed during lockdown, resulting in weight increase [17]. As more individuals have sought ED services, providers have found that people have been spending increased time with family, whether by choice (e.g., choosing to move back in with family) or force (e.g., limited ability to leave home due to lockdown) [6]. As a result, people who cannot escape previous ED stressors are being forced to hide EDs behaviors from family while living at home during lockdown or include family members in their ED-related concerns. They may also be unable to utilize coping mechanisms they have developed independently of their family structure [7, 18].

A lack of control is inherent to a pandemic, and the idea of control has particularly deep roots within ED literature [19, 20]. Those battling EDs have experienced more of a “lack of control” response, and those on the road to recovery had “lack of control” responses develop again due to the uncertainty surrounding the pandemic [21]. Within the context of COVID-19, the overall uncertainty associated with the state of the world is a primary contributing factor to those feeling a “lack of control” [18]. People may believe that disordered eating behaviors are the only way to exert control over the uncertainty and loneliness experienced during the pandemic, particularly when there was a lack of access to mental health care [7, 15]. Food insecurity has also caused an increase in disordered behaviors such as hoarding food and panic-buying [7, 21].

Next, social barriers to support have emerged during the pandemic due to lockdowns and quarantines preventing people from seeking professional care or the support networks they would typically access [7, 10, 18, 26]. COVID-19 has significantly influenced all mental health services, resulting in reduced and altered service availability, as well as a strain to the system due to increased service demands [23]. As a result, relationships between ED patients, family, and staff have been affected by new obstacles, particularly as most patients prefer face-to-face meetings which were often barred [23]. Furthermore, other types of support and medical aid, such as family support and general mental health support, have been difficult to access [6].

Finally, although many individuals have had significant setbacks with their EDs during the pandemic, the experience has been found to have some indirect benefits consisting of individuals seeking support from novel sources [18]. Individuals also utilized this time to work on recovery via self-help books, apps, and other resources easily accessible at home [22]. Some behaviors of EDs decreased, such as binging, purging, and social anxiety [7]. Fernandez-Aranda and colleagues assessed changes among those with EDs in Spain due to lockdown, and individuals with AN had a favorable response to lockdown (regarding treatment and decreasing symptomatology), whereas virtual therapy had an unfavorable response among those with AN (posed more challenges than face-to-face therapy) [31]. Furthermore, despite the changes in treatment, only 20% of ED patients report COVID-19 impacted their overall treatment, and they also were able to utilize a variety of helpful coping skills to supplement lacking treatment [23]. The proliferation of virtual appointments also allowed younger ED patients increased access, and helped increase comfort, although telehealth is not typically an adequate treatment option for people with severe EDs [20, 24].

Online support groups, both formal (i.e., organized by psychological professionals) and informal (i.e., organized in online forums as peer-led groups) have also emerged to fill the treatment gap, and the benefits include improved quality of life and enhanced decision-making [25, 34]. Unlike traditional in-person support groups, online groups can also include caregivers, thus allowing them to request advice and support in real-time and learn more about the illness and how to be an effective caregiver [26]. From mothers to partners to siblings and roommates, the diversification of online support group options has allowed a variety of interested parties and loved ones to seek new avenues to help and support the ED patients in their lives [15, 27].

Existing research has examined these online support venues and found that during the pandemic years, the specific symptoms of EDs change due to the substantial impacts of the virus on day-to-day life [15]. However, this research only examined the impacts in the first two months of the pandemic, and thus did not discuss the long-term effects as seen in online support groups as the pandemic transformed from a flashbulb event to the “new normal.” The present study aims to expand the existing analysis by examining data from the first six months of the pandemic, March 2020-December 2020. These date ranges were strategically selected to examine any differences after six months and due to the holidays.

Thus, our aims are as follows:

1. We aim to understand the long-term impact of COVID-19 on the EDs community outside of a therapeutic context.
2. We aim to explore the often-mixed impact of familial influences on those with EDs, particularly when living situations are in flux and are impacted by factors outside of the patient’s control.

## Methods

### Reddit as a source

Reddit, as a platform, provides unique affordances that benefit this study. Effectively a network of message boards, Reddit is arranged by specific topics, called “subreddits,” which typically focus on a specific theme or idea. Often, different subreddits represent entirely disparate views, such as the difference between a pro-ED subreddit and r/EatingDisorders, in which the participants (“Redditors”) are working towards recovery. These subreddits are moderated such that content can be restricted, topics can be deleted, and individuals can be removed from the Reddit community. In r/EatingDisorders, for example, the Redditor who writes the query sends it first to the moderators, who then determines whether it aligns with the subreddit’s rules (e.g., it does not encourage EDs as a “lifestyle choice”), and—upon approval—post the topic to the subreddit. In this way, the fighting individual’s identity remains anonymous except for the half-dozen selected moderators. It allows an even greater sense of disinhibition than is typically experienced in online spaces, thus a greater degree of honesty [28].

Of the various ED-related subreddits, r/EatingDisorders has the largest number of subscribers (over 48,000); addresses EDs in general rather than one ED precisely (as with r/AnorexiaNervosa); and is not set as private/membership only, thus alleviating some concerns regarding user privacy. Reddit has been used in multiple studies investigating EDs and remains a valuable tool to allow researchers to see into the minds of people with EDs without the possible content filters present within therapeutic settings [29].

The choice of this subreddit also created a more ethical environment for the authors to examine. Although many researchers who use the Internet for data consider screen names to be appropriate pseudonyms, critics have noted that many online monikers can be more easily traced back to an actual person than that person’s full name [30]. The British Psychological Society guidelines on Internet-mediated research support not providing screen names when reporting Internet-based data. The Association of Internet Researchers encourages researchers to consider this when mining the Internet for data that could be regarded as ethically dubious [31]. Moderators of this subreddit were not approached for permission to collect data, because the unique structure of r/EatingDisorders as a fully moderated yet entirely public subreddit allows both researchers and readers to be fully blind to original identities, thus protecting their privacy.

### Sample

To gather our sample data, we searched the r/EatingDisorders subreddit for several pandemic-related search terms, including “COVID,” “pandemic,” “quarantine,” and “coronavirus.” A similar approach was used for a more limited study [15]. Since we wanted to examine the evolution of individuals’ perception of the pandemic as it relates to their EDs, one author initially extracted the relevant posts from three separate periods during 2020. The intention of this method was to expand on existing research by exploring how the challenges faced by people with EDs evolved throughout the first six months of the pandemic [15]. The first period (March to June) represents the initial pandemic, lockdown, and many areas emerging from lockdown and included 175 relevant posts (19 in March, 68 in April, 60 in May, and 28 in June). The second period (August to September) was chosen as a significant time in school-aged people’s lives since their educational situation would likely look different in the new school year. This period included 20 pandemic-related posts (five in August and 15 in September). Finally, the third period (November and December) was chosen due to both the symbolic timeframe and the fact that this was the first holiday season since the start of the pandemic. Specifically, this timeframe reflects half a year of COVID being a daily aspect of life and acknowledges the difficulties that people with EDs typically face during the November and December holidays. This final period included 39 relevant posts (26 from November and 13 from December). We then removed anyone who referenced being under-18 to comply with Institutional Review Board (IRB) privacy recommendations. Using Braun and Clarke’s process for thematic analysis, each of the three authors (i.e., coders with experience in data collection and coding) read the posts, identified basic patterns, and found that they had reached a saturation of ideas within the first 150 posts for the first period [32]. Because there were so few relevant posts for the second and third timeframes, all those posts were examined by each author. Following this, each author independently read all the posts and identified ideas and quotations. Then, we collaborated to condense these ideas into themes with exemplar quotations. Any disagreements were discussed in detail until all parties came to consensus. Finally, we each chose a timeframe to document, while the other two authors reviewed the others’ work, contributed feedback, and provided edits.

## Results

Others have explored the themes dominating the first three months of the pandemic, including changes in ED symptoms and changes to the environment (e.g., returning to live with parents and difficulties seeking/receiving treatment) [15]. However, we were more interested in the themes present throughout the pandemic, as well as how those issues changed during the three timeframes chosen. Looking at this broader dataset, we found two themes, a “lack of control” and “familial influence,” that were present in all three timeframes. However, there were several themes present in only one or two of the time periods that speak to individuals’ personal evolutions with regards to the pandemic.

### Theme 1: Lack of Control

Redditors within r/EatingDisorders mentioned that a persistent, ongoing feeling of a “lack of control” permeated their lives due to both internal and external factors.

However, because of quarantine, I can’t stop thinking about food. I’m terrified that I wont be able to control myself in the future… since Quarantine started I have lost pounds I’ll be honest I’m very happy with that but I’m very scale obsessive and very neurotic and I feel like I’m doing this as a way to control a situation that’s out of control. (Time 1)

i’m relapsing badly for the first time in a few years… it’s the only thing i feel in control of anymore. being socially isolated for so long and not knowing what’s going to happen with covid has completely decimated my mental health (Time 1)

I've noticed recently that my brother, who has been worried by a bit of the extra weight he has put on in lockdown, has begun to develop some unhealthy ideas about food, and wanted to ask how I best support him and help him in the right direction… I talked with him and he said he wanted to lose weight [and] to control his calorific intake…he seemed completely fixated with the idea of calorie counting. (Time 2)

Redditors such as these point to food and weight control to control some small part of the larger situation that they find themselves in every day (i.e., COVID-19). Similarly, changes in living arrangements and surveillance (e.g., family members watching their eating behaviors and themselves observing the eating behaviors of family members) also impacted the Redditors’ recovery process. As a result, they felt a “lack of control” due to the uncertainty associated with the length of time they must remain in the new living situation and being under constant surveillance, resulting in a reliance on ED behaviors as a coping mechanism for the “lack of control.”

They also mentioned the overall uncertainty related to the treatment options (and lack thereof) available and skepticism that those options would be accessible. Redditors reported that the closure of businesses, such as restaurants and gyms, impacted their recovery process (i.e., coping mechanisms and outlets). As a result, they felt there was a “lack of control” due to the uncertainty and absence of their typical external avenues of coping, which resulted in ED behaviors increasing or remerging.

However, some Redditors utilized lock-down to “take back” control of their EDs due to having more time to self-reflect. Additionally, some individuals shared that going back home and being under constant surveillance had significant benefits. Specifically, since they knew they were being watched/encouraged by their family, they were able to ignore the EDs’ “voices” and helped them wrestle back “control” of their behaviors. Moving forward, researchers should further investigate the “lack of control” and “control” themes among those with EDs in relation to how these responses have been impacted by COVID-19, as well as ways to help provide individuals with more effective and beneficial coping mechanisms that are less dependent on external availability (e.g., a restaurant being open, or a particular person being available).

As the pandemic progressed, the dialogue around control moved from feeling like their ED symptoms gave them control in an uncontrollable situation to the symptoms themselves escalating “out of control.”

Come pandemic, I finally found the strength to sort myself out…I've been doing great. Come Wednesday - my cheat day. Overeaten. Fine, will start again the next day. As usual. Just this time I didn't. For four days straight I've been stuffing my face with literally everything in my sight, purged 2 times and I am so scared of going back to my old ways... I feel like I've lost control and I do not even have an idea why. It's just like... A switch went off in my head. (Time 1)

I’m a freshmen in college, but have had these thoughts since my sophomore year of high school. I did exercise almost obsessively but school tended to keep me occupied enough to keep my mind off of food. However, it has gotten significantly worse since quarantine started in March. Some days I feel like I’m spiraling out of control and others I feel like I’m making this all up in my head and nothing is wrong with me. (Time 2)

Redditors also discussed how they had experienced stress and anxiety due to being essential workers. As a result, these users felt a “lack of control” due to the increased levels of stress and anxiety at work, which can result in their ED behaviors becoming more deeply entrenched or reemerging.

This is my first time posting here. Prior to quarantine I had finally stopped all binge eating and the last six months I have lost almost a good amount of weight healthily. I also am a nurse at a hospital and with COVID, I have become even more depressed watching people die and staff also get sick. This is now manifesting into me restricting everything. I feel guilty for eating anything now; before it was anything unhealthy but I was trying really hard not to restrict, only set healthy limits. Now, it doesn’t matter what it is, if I eat anything I just feel guilty and worthless. (Time 1)

Finally, some also discussed the implications of having contracted COVID-19 on their ED symptoms. For example, the resulting symptoms such as decreased sense of taste and smell impacted their overall appetite.

I came down with covid and completely lost my appetite. I was eating one meal a day and it was small. I was trying to eat more, but I was full and had no interest. I also completely lost my sense of taste and smell, and that made things harder, too. Now, I am mostly recovered. I still have low energy and some breathing problems (but it sounds like it might take months for that to be 100%). Anyway, I find myself still restricting a lot. My appetite is kind of back, but I really can't tell if its me restricting or that it hasn't come back. (Time 1)

In addition to the practical effect of reducing one’s desire to eat and eliminating the sensory pleasure of consuming food, people found they could no longer trust their bodies to be healthy, functional, and let them know when they needed to eat. As a result, they felt a “lack of control” regarding regaining their previous appetite, and thus felt more in danger of relapsing.

### Theme 2: Familial Influences (loved ones seeking support)

Another essential element to note is the effects of ongoing confinement on being exposed to people and behaviors that influenced the initial development of disordered eating patterns. Despite a lack of empirical evidence that families are a negative influence prior and during the recovery process, Redditors who mentioned their families in their posts rarely saw them as positive forces in their recovery [33]. Instead, families were perceived as a direct cause of the individual’s ED. Furthermore, those whose families were not seen as directly contributing to the user’s ED were often seen as engaging in triggering behaviors, such as their own disordered eating, surveillance/monitoring of their child’s eating, and general criticism.

But my family really exacerbates my ED. Whenever I eat, I am policed… My dad especially gets bad. Now I’m eating a mix of like, 5 foods that he’s “approved” because I don’t even want to deal with the screaming match that will ensue if I eat something “bad” but damn..This just fucking sucks. I should be better, my dad shouldn’t be such a psycho, I shouldn’t be doing this, and I hate the fact that I’m at home and not in control of my life again. (Time 3)

Negative “family influences” seem particularly harmful when the user loses access to other support system elements (e.g., psychological help, friend groups, healthy routine). The confluence of familial stressors and lack of traditional coping mechanisms sets the stage for the potential of relapse or an increase in disordered eating behaviors. As the pandemic progressed and people remained in problematic living situations, they felt the familial stressors worsen, particularly as some individuals have been home with triggering family members since the pandemic began.

So now it’s September and my mother has been continuously making comments about her own weight...since the pandemic has broken out... it’s triggering to me…[for] a while I was able to brush off the comments but now reaching 6 months of hearing these comments almost everyday I’m start[ing] to break. (Time 2)

However, living alone during lockdown was not a good solution for many, as several users reported intense feelings of loneliness. Because the act of eating is often tied to social events, being forced into social isolation for long periods of time challenged people who struggle with EDs, as many simply do not eat when alone, or their eating habits fundamentally change when not observed and held accountable.

On the other hand I always eat all my food when I am with other people like my friends but when I am alone I just don’t eat. (Time 3)

A subtheme that emerged was “loved ones seeking support.” In other words, individuals used r/EatingDisorders to seek advice on how to support a loved one battling EDs. Stay-at-home orders increased the time that family members spent together, allowing them to observe each other’s behavior. As a result, some individuals learned either that their loved one has an ED or noticed that they were engaging in more ED behaviors than usual. In these instances, users turned to the subreddit to ask how they can best support their loved ones, especially when professional help is less accessible due to COVID-19.

I have a child (16trans) that is dealing with an eating disorder. I have a couple questions about how you would want your parents to approach a ED…I just want to support our teen, but I don't know how to react when we're told one thing by doctors and another by our teen. (Time 3)

Although we saw the broad familial theme most often in the context of people with EDs returning home to their parents and confronting issues, we also saw that many Redditors were concerned about newly observed behaviors of family members, most often mothers and sisters.

My twin sister wants to lose weight, but tracking calories makes her feel very uncomfortable…I suspect her eating is somewhat disordered. She’s my best friend and I want to do right by her… What language or methods do you use? I want to help her be her best self, but I don’t want to force the concept of calorie counting when it’s clear it will impact her mind negatively. (Time 3)

Fast forward to now, [my sister] is back home because of COVID quarantine and I can tell she’s not eating enough (Time 1)

We just found out [my sister] struggles with [disordered eating] tonight. I’m home from college because of COVID and found her throwing up because she hadn’t eaten all day and was using drugs… to try to lose weight (Time 1)

As the COVID-19 crisis becomes less acute and clinicians begin to see new patients, they may observe an increase in ED patients simply due to previously absent family members returning home and intervening with or influencing their behavior.

In addition to these two themes that permeated all three collection periods, we found several themes that were significant, but only present in one or two of the collection periods. We will now detail those.

### Theme 3: Symptom Stressors

As the pandemic progressed, Redditors had significant concerns about a variety of different ED symptom-related issues. Concerns related to weight gain and loss often dominated the discourse. As individuals ended their lockdowns, they experienced the added stress of seeing others for the first time after weight changes, and those who experienced changes in their weight during quarantine encountered negative comments about their body from friends that they had not seen since Covid began.

I’ve been pretty overweight my entire life and felt quite insecure about it. When quarantine in my country started, I decided to pick up running...I started to notice I had a problem because I would make myself feel extremely guilty for missing a workout...Or if I ate 2 burgers instead of one I would have to do a run...Since I haven’t seen my friends in a while (due to lockdown) they were all shocked at the complete body transformation I have undergone. One person even put their fingers around my wrist and said “ur too skinny.” (Time 3)

As people adapted to the “new normal” of the pandemic, they reflected on how lock-down had affected their thoughts and behaviors related to eating and body image. Some individuals who were recovered from an ED when lock-down began found themselves relapsing due to too much free time or a “lack of control.” Sometimes, what began as people trying to get in shape during lockdown became new ED symptoms that they worried were spiraling “out of control.”

I’ve noticed that I often feel panicked when I think about making/getting/eating food. I can't decide what I want to eat [be]cause of the stress so I tell myself that it’s ok to not eat at all. I think the feelings could be stemming from a lack of control due to covid.

But quarantine came. I became OBSESSED with food an exercise, once again. Counting every single calorie, exercising two hours everyday. I had SO MUCH FREE TIME. It consumed my life. (Time 2)

Other users began experiencing ED thoughts and behaviors for the first-time during lock-down.

I have a night eating disorder really bad. I have no appetite during the day AT ALL...But at 2am, I get an appetite and want to eat...it usually gets me binging junk food...What’s weird is this had been going on since Covid. (Time 3)

I’ve always been a little on the overweight side...Until lockdown happened and I, like many, used the extra time and energy to motivate myself and achieve my “dream body.” Now 6 months down the road I fear what the future has in store for me. (Time 3)

On the other hand, some users who had not seen loved ones during lockdown expressed concerns about others’ appearance changes. Furthermore, those with loved ones who had a history of EDs expressed concerns about weight changes during the lockdown, along with the negative impacts on mental health.

We [my friend and I] didn’t see much of each other due to schedules / Covid. Recently she [my friend] accepted a job out of state...and swung by the house to say goodbye. She looked skeletal, and ill. Myself and the rest of my household noticed immediately...I’m not sure how to bring up that we’re worried about her in a delicate way. (Time 2)

She [my friend] reached out to me recently in the hopes that I “keep her accountable” since she gained some weight during COVID...Last weekend was supposed to be her wedding, but she said she didn’t feel “skinny enough” so she’s pushing it to “whenever I lose xx pounds.” (Time 3)

These differences in appearance also support current changes present due to variations in technology reliance, which is the next theme we found.

### Theme 4: Technical Stressors and Concerns

Next, people took note of the unique challenges posed by attending school virtually. First, individuals indicated that in-person school used to serve as a distraction from their ED thoughts and behaviors, but online classes fail to fulfill that need. Because online classes lack the accountability of face-to-face courses, Reddit users found it easier to neglect school to focus on their ED.

It’s [my ED] taken a really big toll on my mental health and online schooling this past month; I’d do sit-ups non stop during live sessions or think of how to get out of eating a lot at dinner (Time 3)

Since the pandemic started and I’ve been having online uni classes...I’m neglecting school responsibilities because of my ED. When I had to go to school, I could devote my time to attend classes and study in the library...Worrying about school meant less time I spent worrying about my ED. Now I spend 24/7 in my home, I don’t have anything separating my free time from my school time, and I spend all day thinking about what I’ve eaten, what I shouldn’t eat tomorrow, how much exercise I should be doing later, etc (Time 3)

Another complication posed by online classes was the expectation (or requirement) to turn on web cameras. Individuals were hesitant to turn on their cameras because they did not want to see themselves and were embarrassed to be seen by their classmates.

I don’t even want to do school tmrw because I’m going to have to turn my camera on and I don’t want anyone to see me. (Time 3)

My self-esteem and confidence is at it’s lowest to the point where I would not open my cam during classes. (Time 3)

The transition to exclusively online therapy also posed challenges, both for the reasons and the perception that telehealth care for EDs was less effective than in-person healthcare. This is particularly relevant when group therapy and in-patient treatment options are removed, as those can be challenging to replicate in online environments. Some users also reported that telehealth was simply not covered by their insurance providers, and thus they were turning to Reddit for the help they would otherwise seek from their professional provider. Typically, people expressed that they would seek treatment in traditional medical establishments, but they were prevented from doing so because of COVID and associated shutdowns. The lack of psychological care exacerbated their anxiety and resulted in them turning to r/EatingDisorders for the support they would typically seek for themselves or their loved ones. Some noted that although treatment was available in the form of telehealth that replacement was perceived as less useful.

I know that the Internet can’t diagnose me, but right now with the quarantine I can’t go see anyone and my insurance doesn’t pay for virtual therapy, so I was hoping this community would be able to at least point me in the right direction or give me advice?

My therapist and I have decided to look into virtual treatment options for me, since in-person can’t be an option right now...Obviously typical in-person treatment is better but has the virtual stuff helped at all?...I feel that virtual treatment won’t even be worth it because there’s so much that can’t be the same (Time 1)

This perception is partially supported by research, indicating that people with EDs face challenges in virtual environments because the medium requires them to see themselves simultaneously while communicating with their health provider [34], resulting in individuals critiquing their image more [7]. In fact, “zoom dysmorphia” is becoming more frequent among those that struggle with their overall appearance [35]. Other concerned individuals noted that a lack of privacy meant that they could not fully embrace the therapeutic relationship and thus did not expect telehealth to be effective. However, a meta-analysis of telehealth efficacy has reported that online telehealth options were equivalent to in-person interventions, indicating that these concerns may be related to anxiety around change rather than an actual failure [36].

### Theme 5: Silver Linings

The most surprising element we uncovered was that early on many people saw the pandemic as having a silver lining. Specifically, self-reflection allowed some users to realize that they have an ED, prompting them to seek advice and treatment. Additionally, some were able to use the time to focus on recovery and take more sustainable steps towards eating food consistently and having regular exercise routines.

Since we’ve been in quarantine I’ve been alone with my thoughts for long periods of time and this has led me to self reflect. I’ve started to come to the conclusion that I might be suffering from an eating disorder and wondering if I should seek help? (Time 1)

I’ve been waiting to have a long period of time to recover, and I took quarantine as an opportunity (Time 1)

Although relatively few people identified the pandemic as a beneficial force in their lives in the two later timeframes examined, the reduction in posts referencing COVID and COVID-related issues from the first timeframe to the subsequent times suggests a building of resilience throughout the pandemic as people began adjusting to the new normal.

## Discussion

This study sought to understand the impact of COVID-19 on individuals fighting EDs. Notably, this research explored how Reddit users discussed COVID-19 and EDs on the subreddit r/EatingDisorders at three critical points in the first nine months of the pandemic. This study's findings can help clinicians provide the best possible services and help those experiencing similar challenges (i.e., EDs) by providing beneficial information [3, 37]. Experiences found within this study consisted of current treatment plans, change of routine and living situations, isolation, COVID-induced stress, and physical bodily changes —all of which are supported by recent research (per our literature review). The results of this study lead to new considerations and practical implications for those managing ED’s, EDs health practitioners, and EDs caregivers.

### Practical recommendations

Because of the information revealed in this study, we have numerous recommendations that we have divided by groups of individuals impacted by EDs: (1) those battling EDs and body image issues, (2) loved ones of those fighting EDs and body image issues, and finally (3) the health practitioners and health promoters who have patients diagnosed with EDs.

#### Those battling eds and body image issues

Those battling EDs and body image issues can benefit from limiting or reframing their media use, as well as attending local support groups and seeking treatment options. In terms of limiting media use, individuals fighting EDs can, and in some cases *should*, limit or eliminate potentially triggering social media accounts, such as those focused on losing weight or changing one’s appearance. In terms of reframing media use, we encourage individuals to remind themselves that most images seen online are published through a filter or manipulated via image-editing software [37, 38]. Therefore, the images that patients are typically seeing do not represent an achievably reality, and thus should not be modeled. In addition to these steps, seeking some variety of social support is critical to recovery. Local support groups and treatment options are likely the superior options, as individuals will be exposed to others that can help via personal experience, empathy, and expertise. However, even virtual support groups—such as those that proliferated with COVID-19—and online venues—such as r/EatingDisorders—will provide them with solidarity, social interaction, and support. They should exercise caution when joining online groups, however, as some groups believe that EDs are positive or beneficial, and thus will not meet the needs of a person seeking support that is focused on recovery [39].

Limitation and reframing of media technology for those battling EDs is especially persistent in a post-COVID-19 world since most institutions transitioned to distance learning in Spring 2020. There has been debate among educators regarding camera use in synchronous online classes. For example, 77% of K-12 teachers in the United States require that students leave their cameras on during class because visual contact strengthens engagement and social relationships while allowing online learning to more closely mirror a traditional classroom; critics note that requiring camera use can be culturally insensitive, racist, sexist, and classist [37, 38, 40]. Although most students leave their cameras off due to concerns with their personal appearance, amongst people with mental health issues, the effects of so-called “zoom dysmorphia” are magnified [33, 41]. Our results support that people with EDs are particularly negatively affected by required camera usage and provide further evidence that camera use during distance learning should be encouraged but optional. If videos must be on for some reason (for example, if someone were being interviewed for a new job or media appearance), people with EDs should consider simply hiding the “self-view” so they cannot see themselves. Additionally, certain software packages allow filters to be applied automatically, which may offset some negative effects of viewing oneself, and allow users to feel a sense of control for their appearance. Finally, companies who make video-chat products (i.e., Zoom, Skype, Apple, etc.) should adjust the settings so that the self-view can be disabled by default.

#### Loved ones of those fighting EDS and body image issues

Just as it is critical for those fighting EDs and body image issues to seek support, so too is it critical that the support system and loved ones of those patients look for support from others in similar situations. Specifically, attending both in-person and virtual support groups that cater to counseling the loved ones of those battling EDs and body image issues can help supporters receive mutual aid and real-time interaction with individuals that have had similar experiences. We saw numerous friends, siblings, and parents looking for support on Reddit; in addition to using online forums, seeking out clinical support groups will benefit from receiving both peer and expert advice from individuals trained within this area to make sure they can effectively and accurately support those they love.

#### Health practitioners and health promoters

The best way that health practitioners and promoters can help those currently fighting EDs is simultaneously the easiest and most challenging: increasing staff, increasing traditional and virtual support and treatments, and increasing online support resources. Although the initial costs of a comprehensive strategy may be out of range for many practices, even taking a small step such as offering a peer-led virtual support group can help reach patients who may otherwise be unable to seek treatment. More robust strategies, such as increased staffing, allows more individuals to be helped and aid to be more individualized. Additionally, with more staff, more perspectives can be incorporated to help provide an array of approaches to help those battling and their loved ones.

Practices should first consider increasing virtual support and treatments, as locating patients that are not geographically proximate can both help offset the initial costs and allow additional treatment options in mental healthcare deserts while increasing the total number of patients helped. For example, many individuals may never have sought treatment prior to COVID, due to the inability to attend in-person support sessions and treatments (e.g., lack of access to transportation, lack of income to pay for travel and treatments, etc.).

Additionally, practitioners and promoters could offer more virtual peer support groups for those fighting as an opportunity to socialize with those that are currently experiencing similar challenges via Zoom, such as “Zoom dysmorphia.” These individuals could always join the virtual peer support groups with their camera off, so they are still able to obtain their much-needed support while breaking down barriers (via seeing oneself constantly) that would otherwise deprive them of this opportunity. Providers may also encourage their patients to use software packages that allow users to choose avatars; although such filters have largely been in the purview of young children and technologically inept attorneys, a clinician could use it as a way of understanding the patient’s evolving body image by using virtual representations of how an individual sees themselves, and perhaps even a novel approach to classic pictorial instruments used to measure body image [42, 43]. Virtual support groups can also include those who have loved ones (i.e., a partner, family member, friend, etc.) who exhibit disordered eating behavior. Like the r/EatingDisorders subreddit, these support groups would both provide a service and—unlike the subreddit—provide live, clinically supervised, and validated feedback, as well as the opportunity to communicate/brainstorm with fewer potential barriers (via misinterpretations) than what may occur via written communication.

We also recommend an increase in virtual appointments for those who are hesitant to attend in-person appointments or do not have access to local, reliable healthcare specializing in EDs and body image issues. In our study, the number of COVID-related posts peaked in April (n = 68) and May (n = 60) when much of the US and UK were in full lockdown. Common concerns in these posts were a lack of treatment options and limited access to support. As areas began opening back up, treatment and support opportunities increased, and these themes did not appear in the Time 2 or Time 3 datasets. Although not directly connected to provider support, many users noted that the telehealth options that were available were not covered by insurance, suggesting a need for either insurance companies to expand their coverage (particularly during local and global crises) and providers to adopt income-based sliding scales for telehealth services.

In their efforts to increase online support resources, health practitioners and promoters can develop materials useful for those fighting EDs and body image issues and their loved ones. Particularly, they should create, promote, and distribute these materials in a way that can be easily accessible and relevant to a variety of populations. These materials can provide another beneficial form of support that can be utilized regardless of the time of day or accessibility of resources and can be distributed by practitioners to help patients in stressful times.

## Limitations

Despite the contributions of this study, some limitations must be addressed. The only social media platform analyzed in this study was Reddit, and therefore it is not a comprehensive representation of all online social media support groups. Future research should continue to explore social support groups for EDs posts on different social media platforms to examine the conversation surrounding the impact of COVID-19 on EDs. Comparative analyses regarding the communication of COVID-19 and EDs among various social media platforms could be conducted to flesh out the understanding more fully. Future research should also examine discussions longitudinally from the beginning of the pandemic in early 2020 to 2021, 2022, and beyond (particularly as COVID-19 shifts to an endemic disease). Finally, in some areas, COVID-19 has become a source of controversy for some because it is not as deadly as other diseases; after the initial lockdown period, many people went back to work and school and simply accepted the risk of contracting the disease because they probably would not die or suffer long-term consequences. However, epidemics and pandemics will doubtless become more common as the world becomes increasingly globalized and interconnected. Therefore, examining the effects of more deadly outbreaks on long-term mental health conditions, such as EDs, would be beneficial.

Next, due to the subreddit posts being online and open access, we could only obtain information posted to the subreddit. In other words, we could not probe further discussion on some comments or ask clarifying questions to ensure we fully understood users’ concerns and their goals in utilizing this platform to seek advice and support. Additionally, we could not obtain demographic data regarding our participants, which could have provided additional context.

## Conclusions

In this paper, we utilized thematic analysis to examine the support-seeking behaviors of the users of the subreddit r/EatingDisorders, specifically as they relate to concerns around COVID-19 over three time periods in 2020. We discovered support for the current (quantitative) literature that people with EDs have had difficulty controlling their present situation, have had to navigate increased familial interaction (both positively and negatively), and have actively been using online forums as a way of seeking social support because their existing support structures have either failed or otherwise become untenable.

The most important implication of this work comes in the post-COVID-19 clinical work. Most notably, the conversations surrounding EDs on r/EatingDisorders have continued as the pandemic remained a part of daily life. In fact, the many conversations emerging in this online space highlight the need for extraneous variables to be addressed (e.g., telehealth appointments). As a result of the pandemic, there is a need for psychological intervention and practitioners should expect to see the long-term effects of people not seeking treatment for their EDs. Furthermore, we can also expect to see entirely new EDs patients coming into treatment as their family members have identified newly emergent EDs patterns.

## Data Availability

The datasets used and/or analyzed during the current study are available from the corresponding author on reasonable request.

## Abbreviations

AN: Anorexia nervosa
ED(s): Eating disorders
COVID-19, COVID: Coronavirus disease 2019
FBT: Family-based treatment
IRB: Institutional Review Board
US: United States
UK: United Kingdom
